# ADHD overdiagnosis and the role of patient gender among Iranian psychiatrists

**DOI:** 10.1186/s12888-021-03525-3

**Published:** 2021-10-19

**Authors:** Ashkan Beheshti, Mira-Lynn Chavanon, Silvia Schneider, Hanna Christiansen

**Affiliations:** 1 Department of Psychology, Child and Adolescent Clinical Psychology Group, Marburg University, Gutenbergstr.18, 35037 Marburg, Germany; 2 Department of Clinical Psychology, Child and Adolescent Psychology, Ruhr University Bochum, Bochum, Germany

**Keywords:** ADHD, Overdiagnosis, Gender differences

## Abstract

**Background:**

Regarding the controversy about the overdiagnosis of Attention Deficit/Hyperactivity Disorder (ADHD) in children and adolescents there are two main directions addressed as issue of age bias and issue of gender bias. In this relation, replication of findings demonstrating significant overdiagnosis is of importance which make the systematic evaluation of such occurrence necessary.

**Objective:**

The seminal study by Bruchmüller, Margraf & Schneider, 2012 is replicated here, although in a different cultural context, in this case Iran, as ADHS might be perceived differently there. We assessed both gender bias and the impact of potential overdiagnosis on treatment recommendations.

**Methods:**

A total of 344 licensed Iranian psychiatrists (mean age = 45.17, SD = 9.50) participated in this study. Each psychiatrist received a cover letter that introduced the study as well as a case vignette. Overall, there are eight different cases, one child with ADHD and three non-ADHD children, for both a boy (Ali) and a girl (Sara). Participants also received a questionnaire requesting their particular diagnosis, treatment recommendation and the therapist’s sociodemographic information. Chi square tests and multiple logistic regression were applied for data analyses.

**Results:**

Overdiagnosis occurred in both girl and boy children, although overdiagnosis was 2.45 more likely in boys than in girls (*p* < 0.01). With respect to the psychiatrist’s gender, we detected no difference between males or females, as both overdiagnosed ADHD in boys (*p*_female_ < 0.01 and *p*_male_ < 0.01). Furthermore, ADHD overdiagnosis had a direct impact on medication prescription (*p* < 0.01).

**Conclusion:**

This study suggests that diagnosticians should strictly adhere to diagnostic criteria to minimize diagnostic error.

**Supplementary Information:**

The online version contains supplementary material available at 10.1186/s12888-021-03525-3.

## Introduction

### ADHD and the debate of overdiagnosis

Attention deficit hyperactivity disorder (ADHD) is one of the most frequently diagnosed disorders of childhood and adolescence, with a pooled worldwide prevalence rate of ~ 5% [1–3]. A meta-regression analysis of 102 studies by Polanczyk et al. [3] demonstrated that variation in methodological diagnostic procedures is considered the main source of variability in prevalence estimates. The three parameters diagnostic criteria, information source (parent ratings vs. teacher ratings vs. clinical interviews), and functional impairment reveal a significant association with the variability of estimates in this context [1]. There are recent studies demonstrating a rise in the rate of ADHD diagnoses. For instance, Xu et al. [4] analyzed data collected by the National Health Interview Survey during the past 20 years and reported an overall ADHD prevalence rate of 10.2% among US children and adolescents aged 4 to 17 years (6.3% in boys and 6.1% in girls) for the years 2015 to 2016. Compared to a rate of 6.1% in the years 1997 to 1998, that is a significant increase for all subgroups [1, 3]. Furthermore, Chung et al. [2019] reported that the prevalence of adult patients aged 25 to 34, who received an ADHD diagnosis increased from 0.43% in 2007 to 0.96% in 2016. Similar analysis for children aged 5 to 11 years showed that prevalence increased from 2.96% in 2007 to 3.74% in 2016. The above-mentioned study is based on the reports of licensed mental health clinicians who diagnosed ADHD for more than five million patients cared for at Kaiser Permanente in Northern California.

Moreover, accompanied by increasing diagnoses rates of ADHD, there are studies that imply a rise in the rate of psychostimulant prescription. For instance, a longitudinal study by McCarthy et al. [5] investigated pharmacological treatment trends for ADHD in the UK. Their study findings imply an increasing rate of medication prescription from 2003 to 2008: they noted an increase in children from 4.8 (95% CI: 4.5–5.1) to 9.2 (95% CI: 8.8–9.6); in adolescents from 3.6 (95% CI: 3.3–3.9) to 7.4 (95% CI: 7.0–7.8); in youth from 0.3 (95% CI: 0.2–0.3) to 1.1 (95% CI: 1.0–1.3); and in adults from 0.02 (95% CI: 0.01–0.03) to 0.08 (95% CI: 0.06–0.10). Another of this study’s findings was that this increase is significantly higher for male than female children, though in adolescence and adulthood this was not the case as the rate was significantly higher for female patients. Bachmann et al. [6] compared the prevalence of ADHD medication use in five countries between 2005 and 2012 and reported an increase from 1.8 to 3.9% in the Netherlands (relative increase: + 111.9%), from 3.3 to 3.7% in the US (rel. Increase + 10.7%), from 1.3 to 2.2% in Germany (rel. Increase + 62.4%), from 0.4 to 1.5% in Denmark (rel. Increase + 302.7%), and from 0.3 to 0.5% in the UK (rel. Increase + 56.6%).

With respect to such diagnosis and prescription rates, on the one hand there are studies suggest that these rates are increased during the past decades due to better recognition in underdiagnosed groups, extended knowledge on psychopathology and diagnostic procedure of the disorder, and increased access to health care in certain groups [7–10]. Therefore, these findings legitimate the increased rate of ADHD diagnosis and medication prescription. On the other hand, there are justifiable concerns about ADHD’s overdiagnosis summarized as issue of age bias and issue of gender bias.

### ADHD overdiagnosis and issue of age bias

A large body of ADHD research shows that children born close to kindergarten or school cut-off dates, and who are therefore up to a year younger than their classmates, are 30 to 60% more likely to be diagnosed with ADHD [11–14]. In this regard, there is evidence that a child’s birthdate influences considerably the subjective evaluations of teachers in identifying whether that child is exhibiting ADHD symptoms. For example, Elder [15] showed that according to school entry dates, the youngest children in a class are 1.6 more likely to receive an ADHD diagnosis than the oldest children in the same class, implying a significant age bias that is confounded with development. This finding was replicated by Morrow et al. [16] in children in British Columbia. Furthermore, Layton et al. [17] similarly compared the rate of ADHD diagnosis between 2007 and 2015 from a large insurance database in all U.S. states for children born in August and who born in September. They reported that rates of diagnosis and treatment were significantly higher among children born in August. Wuppermann, Schwandt, Hering, Schul and Bätzing-Feigenbaum [18] also made such observations in their study in Germany, concluding that there is a robust association between ADHD diagnosis, psychostimulant treatment and relative age position in the class due to month of birth and school entry dates.

### ADHD overdiagnosis and issue of gender bias

Overall, the majority of studies report an ADHD gender bias that puts boys at a disadvantage. The rate of boys to girls diagnosed with ADHD is reported to be 3 to 1 in representative population-based studies [4], and 5 to 1 or 9 to 1 in clinically-based studies [15], and such gender differences may play a significant role in the case of ADHD overdiagnosis [16]. In addition to and regarding the pharmacological treatments for ADHD, there are studies demonstrating that boys are given significantly more medication than girls i.e. 3 to 1 in the US [17], 7 to 1 in the UK [19] and 5 to 1 in western European countries [18]. As reviews suggest, an explanation for such differences between boys and girls in ADHD diagnosis and medication prescription is that symptom manifestation in boys and girls differs in such a way that girls display more inattentive symptoms that are not found to be as disruptive at school or home [20–23], while boys exhibit more hyperactive, impulsive and aggressive behaviors that parents and teachers find to be more burdensome and thus result in higher referral rates [21, 24, 25].

Regardless of the diverse manifestations of ADHD symptoms in boys and girls, another explanation for the different ratios in boys and girls is clinicians’ subjectivity. Bonati and Reale [26] state that although ADHD practice guidelines highlight the assessment of symptom severity based on developmental, medical and psychosocial parameters, the degree of impairment is still partly determined subjectively by the clinician. Mertens, Cwik, Margraf and Schneider [21] suggest that diagnosticians may not be adhering strictly enough to diagnostic criteria and that instead, their clinical judgment is being affected by heuristics and biases. They demonstrate that diagnosticians are prone to making mistakes in the decision-making process. In this relation, on the one hand heuristics are considered to be cognitive shortcuts simplifying the procedure of clinical decision making in uncertain situations [27]. Evaluation of the probabilities is facilitated by the three factors of anchoring, availability, and representativeness. Anchoring refers to decision making based on the single piece of data provided in a primary encounter. Availability is an element of heuristics adopted when the occurrence of an event is more probable due to the more recent or emotionally salient examples. Representativeness refers to a situation when assessment is made with respect to the similarities which makes a case significantly probable to belong to a certain class [28]. On the other hand, although these factors can function as short circuits of decision making in clinical situations, they may introduce bias result in error. In this regard, undervaluing the later information, omission of less distinguished but still considerable data, and too much focus on pattern recognition are sources of possible risks in adopting heuristics [29, 30]. Furthermore, Metta [31] showed that heuristic bias may depend on years of job experience. In this relation, clinicians’ judgment dealt with two core characteristics: (a) the emotional reactions generated during the assessment and (b) a shift in the clinicians’ attitude over the years, prompted by experience and personal change [31]. Adopting a heuristic approach, clinicians tend to base their diagnostic decision primarily on their subjective perception of the patient. With respect to ADHD diagnosis, as patient gender moderates symptom manifestations, boys are more likely to receive an ADHD diagnosis even when not all the criteria have been met (false-positive diagnostic error), while girls are less likely to receive a diagnosis of ADHD even when they fulfill the criteria (false-negative diagnostic error). Therefore, considering the heuristics effect in the diagnostic process is another possible explanation for the differences we observe between clinical and epidemiological data in ADHD [13, 24].

In support of this are results from a meta-analysis that estimated the agreement between diagnoses made from clinical evaluations and standardized diagnostic interviews. Overall, such agreement was low to moderate and depended on the disorder, with ADHD resulting in moderate agreement (kappa = 0.49, CI = [0.46, 0.52]) [32]. However, the studies included in the meta-analysis reported kappas varying from 0.12 to 0.92, indicating that the accordance between ADHD diagnoses derived from clinical evaluation and standardized diagnostic interviews requires further systematic examination.

### Systematic examination of ADHD overdiagnosis

On the other hand, and in order to defend the validity and reliability of diagnoses and treatment by the professional, it is necessary to work through this debate of overdiagnosis of ADHD in terms of associated socio-economic and individual implications [33]. After all, if our field provides interventions, we should also be able to defend our diagnoses. However, without conducting comparative studies that systematically assess the factors involved in overdiagnosing ADHD, potential indicators such as the variation in prevalence or increasing rates of stimulants prescriptions should not be interpreted as indicative of overdiagnosis [9]. In this regard, Sciutto & Eisenberg [34] suggest investigating the occurrence of overdiagnosis by reexamining referred patients and by conducting comprehensive, multimethodical evaluations to be compared with the actual diagnoses. Such arguments address type I or false-positive diagnostic errors. Sciutto and Eisenberg [34] criticize such focusing on false-positive and the disregard of false-negative errors (type II), however. Instead, they suggest examining the presence of potential overdiagnosis via a ratio of these two errors. In this regard, comorbidity, diagnostic inaccuracy and changes in diagnostic criteria are sources of false-positive or type I errors, while gender, cultural norms and barriers to diagnostic assessment and treatment are false-negative or type II errors [34]. Accordingly, ADHD overdiagnosis should be based on the odds ratio of type I to type II error while also considering the significance of this ratio, since to consider the mere increased prevalence rates or rates false positive ADHD as an indicator of ADHD overdiagnosis, lacks a comparison to a reference point.

The application of diagnostic criteria for assessing the false positive and false negative ADHD diagnosis and the specific role of patient gender in therapists’ diagnostic decision making was systemically investigated in a seminal study by Bruchmüller, Margraf and Schneider [21] in a population of licensed German psychotherapists and psychiatrists (*N* = 473). Overall, there were significantly more false-positive than false-negative diagnoses confirming the assumption of overdiagnosis, and boys were given significantly more false-positive diagnoses, thus indicating a gender bias to their disadvantage. This gender bias was boosted by the fact that male psychotherapists and psychiatrists were more likely to falsely rate boys positively than their female counterparts. In addition, Bruchmüller, Margraf and Schneider [21] reported that in vignettes 2–4, those therapists who had made an ADHD diagnosis recommended medication and psychotherapeutic treatments significantly more often than other therapists who had not diagnosed ADHD. They thus provided evidence that ADHD overdiagnosis has a direct impact on treatment recommendation.

Moreover, overdiagnosis of ADHD in children and adolescents is also investigated in a systematic scoping review (including 344 studies) by Kazda et al. [35]. The results of this study approve the occurrence of ADHD overdiagnosis and overtreatment specially for youths with milder or borderline symptoms.

### ADHD in Iran

Aim of the present study was to replicate their study, but based on an Iranian population of psychiatrists. Iran is especially interesting for such a study, as a large survey in 2008 on the multicultural assessment of child and adolescent psychopathology via the Achenbach System of Empirically Based Assessment (ASEBA) and Strength and Difficulties Questionnaire (SDQ) indicated that mean ASEBA and SDQ scores are nearly identical for both genders in large, representative samples speaking 76 languages, except Iran [36]. In this regard, both parents and teachers in nearly all the countries studied other than Iran rated boys higher than girls on DSM-oriented ADHD scales, as well as such gender differences on Attention Problems, Rule-Breaking Behavior, and Aggressive Behavior Syndromes were larger in teachers’ ratings than in parents’ ratings. However, Youth Self Report on ADHD scales revealed no significant gender difference in these societies. In our Iranian sample, girls and boys in Iran attend single-gender schools, and girls in this study were given higher ADHD scores than girls in most other populations, while Iranian boys scored at about the middle of the overall population. Achenbach et al. [36] hypothesize that “the absence of boys in the Iranian girls’ classrooms lowered teachers’ thresholds for endorsing ADHD items in girls, thereby producing higher scale scores than those found in populations whose boys and girls attend class in the same classroom. Unfortunately for this hypothesis, Iranian parents also rated girls as high as boys on statistically-derived and DSM-oriented scales of ADHD problems, unlike parents in the other populations. The similar findings for Iranian parent and teacher ratings indicate cross-informant and cross-situational consistency in Iranian adults’ tendency to rate Iranian girls as high as boys on ADHD problems” [30]. Furthermore, a recent meta-analysis of 27 studies (*N* = 15,124) for the years of 2001 to 2016 reported a prevalence rate of ADHD of 12% (CI 95%: 9.0–15) for children aged six to 14 years in Iran [37]. Compared to the aforementioned international rates [1, 3, 4], that rate is significantly higher. Taken together, these studies’ results make Iran a particularly interesting country in which to investigate the potential overdiagnosis and gender effects of ADHD.

The current study thus addresses the following questions: 1) Is ADHD overdiagnosed in Iran? 2) Is the gender bias to boys’ disadvantage not confirmed in Iran? 3) Do Iranian psychiatrists reveal a gender bias when diagnosing boys and girls with ADHD? 4) Does ADHD overdiagnosis have an impact on treatment recommendations?

In this relation a priori hypotheses of this study are:

1. ADHD is overdiagnosed in Iran.
2. It is hypothesized that Iranian psychiatrists reveal a gender bias when diagnosing boys and girls with ADHD.
3. It is hypothesized that ADHD overdiagnosis have a direct impact on treatment recommendations.

## Materials and methods

### Participants

Among 367 participants of the study, 23 of them were excluded as essential data (e. g. diagnosis or treatment recommendations) were missing. Therefore, a total of 344 licensed Iranian psychiatrists (mean age = 45.17, SD = 9.50) considered to be included in this study, among them 165 individuals from Isfahan and 179 individuals from Tehran (see Tables 1 & 2).

**Table 1:** Demographics of psychiatrists

	N	%	Mean	SD
**Isfahan**	165	47.96		
Age			46.14	10.02
female	102			
male	84			
Years of job experience			15.48	8.83
**Teheran**				
Age	179	52.03	44.02	8.89
female	71			
male	87			
Years of job experience			14.57	7.33
**Overall Demographics**	344	100		
Age			45.17	9.50
female	173	50.3		
male	171	49.7		
Years of job experience			14.53	9.17

Note. SD = Standard Deviation

**Table 2:** Demographics of the cities *

	N	%
Isfahan		
population	2,243,249	
Children (0–13)	57,931	2.58
Parental education		
Primary school or below		27.7
Secondary school		48.8
Academic Education		23.1
socio-economic status		
Upper Class		22.23
Middle Class		45.15
Lower Class		32.62
Tehran		
population	8,693,706	
Children (0–13)	1,516,552	17.44
Parental education		
Primary school or below		21.8
Secondary school		50.6
Academic Education		27.5
socio-economic status		
Upper Class		20.57
Middle Class		39.2
Lower Class		40.23

* Based on publications of General Population and Housing Census of Iran (2019)

we ended up with a final sample of 463 respondents. In contrast to Germany, where licensed psychotherapists and psychiatrists are legitimized to diagnose children with ADHD, Iran only allows psychiatrists to make a diagnosis following the Islamic Republic of Iran’s Medical Council law. We therefore recruited our sample of psychiatrists in Isfahan by twice visiting them in their monthly group gatherings (with a participation mean of 240 individuals) and by distributing the case vignettes, including a cover letter and questionnaire (for details on the study procedure see below). In Tehran we used the database of psychiatrists indexed on the Website of the Iranian Psychiatry Association (IPA) and the documents were sent to 300 psychiatrists’ offices. Among the receivers, 179 psychiatrists sent back their ratings via Post, e-mail or telegram. The data collected between 2019 and 2020. All the participants received and signed ethical consents articulated in a session with the committee of the psychiatric association. We assume the overall response rate to be 44.10% (2 meetings of 240 individuals in Isfahan = 480 + 300 documents sent out to psychiatrists in Teheran = 780 with overall 344 responses) and thus similar to that in the Bruchmüller, Margranf and Schneider study [21].

### Material

#### Cover letter

The cover letter included a brief introduction of this study. In addition, psychiatrists were asked to read a case vignette and answer the questionnaire. We emphasized that despite differences between the fictional case and a real case, they as therapists should treat the case as if it were a real setting.

#### Case vignettes

The four case vignettes already used and discussed with respect to their reliability and validity by Bruchmüller, Margraf and Schneider [21] were also applied in this study. Each case is constructed based on the ICD-10 and DSM-IV criteria for ADHD. Thus, an ADHD diagnosis requires a) six symptoms of inattention and six symptoms of hyperactivity/impulsivity, b) onset before the age of seven years, c) impairment in two or more settings as well as clinically significant impairments, and d) symptoms not better accounted for by another disorder. Finally, vignettes were translated into Farsi and reviewed by four Iranian clinicians to ensure their validity for use in the Iranian psychiatrist community. The case vignettes and questionnaire are reviewed in an established expert committee of Iranian psychiatrists, who are familiar with the construct of interest. Froward and backward translations were provided and where necessary discussed with one of the developers of the original vignettes and questionnaires. Finally, we reached a consensus on all items to produce a prefinal version of the translated vignettes and questionnaire.

##### Vignette 1

ADHD fulfilled: includes a description of a youth with ADHD who presents all the above-mentioned criteria necessary to diagnose ADHD (combined type) based on ICD-10 and DSM-IV.

##### Vignette 2

no ADHD, two criteria missing: The only difference between this one and vignette 1 is that in this case, the youth’s description fails to meet criteria b and c based on DSM-IV and criteria b based on DSM 5. In this regard, the symptoms were apparent in only one setting (school) with the disorder onset after the age of nine years. Therefore, an ADHD diagnosis is not possible relying on either ICD-10 or DSM-IV criteria.

##### Vignette 3

no ADHD, three criteria missing: In this case the youth’s description is similar to vignette 2’s, although a third diagnostic criteria was absent. Regarding criterion a, only two hyperactivity/impulsivity symptoms and three inattention symptoms were present. Thus, although this case still contains some aspects of core ADHD features, an ADHD diagnosis is not justified.

##### Vignette 4

no ADHD, GAD with symptom overlap: As ADHD symptoms and generalized anxiety disorder (GAD) in childhood/adolescence can overlap, this case was constructed to include restlessness, nervousness, and difficulty concentrating, all of which are manifestations common to both ADHD and GAD. However, these symptoms do not suffice to diagnose ADHD, but rather GAD.

#### Gender variation

As one of the aims of the present study was to test whether the patient’s gender moderates a clinician’s evaluation and diagnosis, the four vignettes were duplicated for boys and girls as in the original study to arrive at 4 vignettes for girls and 4 vignettes for boys. The only difference between the descriptions of boys and girls is that the youth in the girls’ cases is introduced as ‘Sara’, a typical Persian name for girls, and the youth in boys’ cases is called ‘Ali’, a typical Persian name for boys.

After duplicating the case vignettes based on the gender there were overall eight vignettes and each psychiatrist randomly received only one of them (between subject design).

#### Questionnaire

Attached to the case vignette, all clinicians received a questionnaire that asked for diagnosis, treatment recommendation and their sociodemographic information. In the diagnosis section, psychiatrists were asked to base their diagnosis of the case vignette on DSM-IV criteria, as DSM-IV is the standard diagnostic manual used by mental health experts in Iran.

In addition, clinicians were asked whether they would recommend any intervention at all (yes or no), psychotherapy (yes or no) or medical treatment (yes or no).

Furthermore, we collected sociodemographic data such as gender, age and years of job experience regarding the therapist’s attributes. We excluded their therapeutic approach as well as the type of therapy training from the original questionnaire as participants were only psychiatrists. In addition, we asked as how helpful they perceived the DSM-IV criteria to be their professional routine (on a scale from 0 means not at all, to 100 means very much), and to estimate their familiarity with the DSM-IV (on a scale from 0 means vaguely familiarity to 100 means very familiar). Moreover, Informed consent was obtained from all individual participants included in the study. Additional informed consent was obtained from all individual participants for whom identifying information is included in this article.

The English version of questionnaire and case vignettes are presented in appendix.1 and 2.

### Statistical analysis

With respect to the first aim of the current study to determine whether ADHD is overdiagnosed in Iran, the percentage of ADHD diagnosis in all cases was calculated according to Sciutto and Eisenberg [34]. In this regard, the occurrence of overdiagnosis is identified when the number of false-positive diagnosis is significantly larger than the number of false-negative diagnosis. Thus, we applied the chi-square test to compare the proportion of ADHD diagnosis in non-ADHD cases (false positive) to the proportion of non-ADHD diagnosis in the ADHD cases (false negative). Moreover, with respect to our second study aim, we applied multiple logistic regression analysis to evaluate the role of gender in the therapist’s diagnostic decision. For this analysis we labeled the youth’s gender (boy vs girl) as the independent variable and diagnosis (ADHD vs non-ADHD) as the dependent variable. Furthermore, to investigate the effect of the psychiatrist’s gender, age, therapeutic approach and years of job experience, we included those factors in the analysis. Finally, we repeated calculations regarding the overdiagnosis separately for male and female therapists. Moreover, by running chi square tests, we compared the treatment recommendations by those therapists who made an ADHD diagnosis in vignettes 2–4 with treatment recommendations by those therapists who did not diagnose ADHD.

## Results

### Overdiagnosis of ADHD

**Fig. 1:**
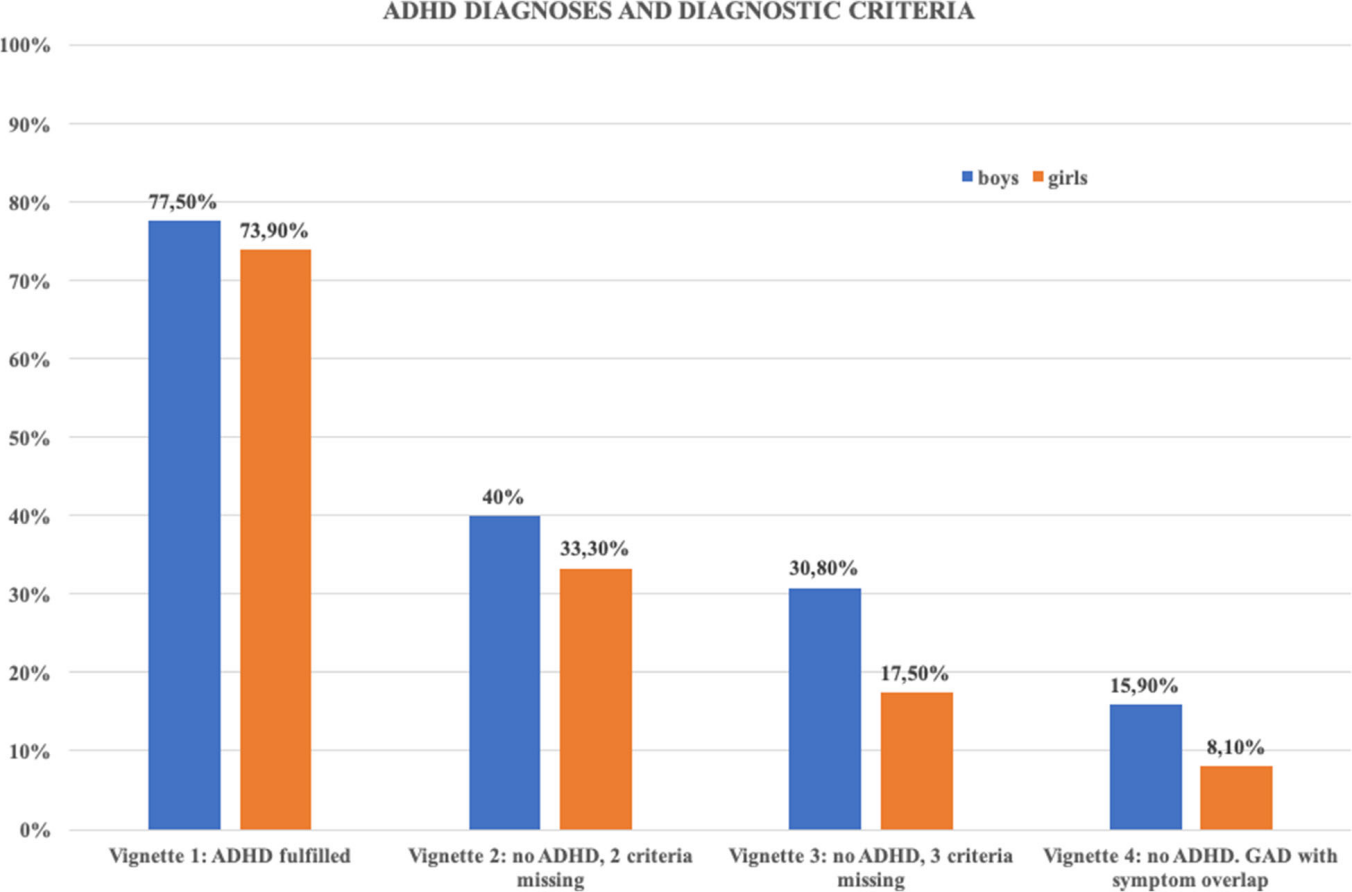
Percentage of attention-deficit/hyperactivity disorder (ADHD) diagnoses for the eight different case vignettes. GAD = generalized anxiety disorder

Psychiatrists’ information on age, gender and years of job experience are summarized in Tables 1 & 2 for both the cities of Isfahan and Tehran. There is no significant difference in the two cities’ demographics (age: t = 1.95, df = 342, *p* = 0.132; years of job experience: t = 1.23, df = 342, *p* = 0.24). Confirming our first hypothesis (overdiagnosis of ADHD) descriptive analysis of all six non-ADHD cases (Vignettes 2–4 with Ali and Sara) showed that 25.2% (*n* = 65) of therapists diagnosed ADHD among these vignettes, while 51.5% (*n* = 133) diagnosed another disorder, and 6.6% (*n* = 17) made no diagnosis at all (see Fig. 1 and Table 3). Moreover, a group of therapists (11.2%, *n* = 29) required more information to arrive at a diagnosis, and 5.4% (*n* = 14) asserted a “suspected ADHD”.

**Table 3:** Diagnoses Given by Therapists in the Different Case Vignettes

Diagnosis	1		2		3		4		Sum 2–4	
	*N*	*%*	*N*	*%*	*N*	*%*	*N*	*%*	*N*	*%*
Results for vignettes featuring a boy										
ADHD	23	57,5	20	40	12	30,8	7	15,9	39	29,3
Other diagnosis	2	5	20	40	17	43,6	28	63,6	65	48,9
No diagnosis	0	0,0	3	6	4	10,3	1	2,3	8	6,0
Not enough information	12	30	5	10	4	10,3	5	11,4	14	10,5
Suspected ADHD	3	7,5	2	4	2	5,1	3	6,8	7	5,3
Sum	40	100	50	100	39	100	44	100	133	100
Results for vignettes featuring a girl										
ADHD	34	73,9	16	33,3	7	17,5	3	8,1	26	20,8
Other diagnosis	3	6,5	22	45,8	21	52,5	25	67,6	68	54,4
No diagnosis	0	0,0	3	6,3	5	12,5	1	2,7	9	7,2
Not enough information	8	17,4	4	8,3	4	10,0	7	18,9	15	12,0
Suspected ADHD	1	2,2	3	6,3	3	7,5	1	2,7	7	5,6
Sum	46	100	48	100	40	100	37	100	125	100
Sum of the results for vignettes featuring a girl and vignettes featuring a boy										
ADHD	57	66,3	36	36,7	19	24,1	10	12,3	65	25,2
Other diagnosis	5	5,8	42	42,9	38	48,1	53	65,4	133	51,6
No diagnosis	0	0,0	6	6,1	9	11,4	2	2,5	17	6,6
Not enough information	20	23,3	9	9,2	8	10,1	12	14,8	29	11,2
Suspected ADHD	4	4,7	5	5,1	5	6,3	4	4,9	14	5,4
Sum	86	100	98	100	79	100	81	100	258	100

Note. ADHD = attention-deficit/hyperactivity disorder; No diagnosis = answers of therapists in the category “no diagnosis for Sara/Ali”; Not enough information = answers of therapists in the category “I have not enough information for already making a diagnosis”

Regarding ADHD cases (Vignette 1) 66.3% (*n* = 57) of the psychiatrists arrived at an ADHD diagnosis, whereas 5.8% (*n* = 5) designated another diagnosis. Besides, 34.5% (*n* = 20) wanted more information to make a diagnosis, and 1.6% (*n* = 4) suspected a case of ADHD.

We then compared the rate of false-positive diagnoses (diagnosis of ADHD in non-ADHD cases) to the rate of false-negative diagnoses (diagnosis other than ADHD in ADHD cases). Overall, 65 out of 258 were ADHD diagnoses in non-ADHD cases (false-positive) and 5 out of 86 were diagnoses other than ADHD in ADHD cases (false-negative). The odds ratio of overdiagnosis amounted to 5.46. In addition, chi square analysis results confirmed a substantial difference between the non-ADHD and ADHD vignettes (X2 (1, *N* = 344) = 14.91, *p* < 0.01). This suggests that the rate of false-positive diagnosis (ADHD diagnosis in non-ADHD cases) is significantly higher than the rate of false-negative diagnosis (not diagnosing ADHD in ADHD cases), thus implying an overdiagnosis of ADHD. Post hoc comparisons of rates of receiving an ADHD diagnosis by with regard to categories of case vignettes (1–4) revealed that significantly higher rates of ADHD diagnosis were seen among case vignette 1 and 2. In comparison, prevalence of no-ADHD diagnoses or diagnoses other than ADHD, was statistically similar among case vignette 3 and 4.

As mentioned above, there were psychiatrists in both groups of vignettes (*n* = 43) who stated no clear diagnosis due to a lack of information, or asserted a suspected diagnosis. In our primary calculation, we considered those answers as neither false-positive nor false-negative, but correct ones. However, this might affect our results, because it is still unclear what their final decision would have been in those cases. Therefore, in our second analysis, we excluded those answers from our calculations and re-examined our first hypothesis. In this regard, the results still indicating higher false-positive rates than false-negative ones, thus confirming the overdiagnosis of ADHD.

Furthermore, besides the ADHD diagnosis, we wanted to discriminate and compare different types of diagnoses other than ADHD that were made in non-ADHD cases. In this regard, our results show that in vignettes 1 and 2, adjustment disorder was the most likely diagnosed disorder with a probability of 0.76 and 0.79, respectively. Moreover, generalized anxiety disorder (GAD) with a probability of 0.83 was the most frequent diagnosis in vignette 4 (see Table 4).

**Table 4:** Diagnoses other than ADHD Given by Therapists in the Different Case Vignettes

Diagnosis	1	2	3	4
	*%*	*%*	*%*	*%*
adjustment disorder	76	79	60	0
disorder not otherwise specified	23	20	36	7
GAD	0	0	0	83
Anxiety spectrum other than GAD	0	0	3	9

Note. ADHD = attention-deficit/hyperactivity disorder; GAD = Generalized Anxiety Disorder

### Diagnosis of ADHD in girl versus boy vignettes

With regard to our second hypothesis, we wanted to explore whether a positive gender bias to the disadvantage of boys is present in Iran or not, as suggested by the Achenbach et al. [36] study. A logistic regression analysis was conducted to examine whether the gender in vignettes predicts an ADHD diagnosis. We also defined the version of the vignette (1–4), gender, age, years of job experience of the psychiatrist, as well as their opinion about familiarity with DSM-IV and helpfulness of the DSM-IV as predicting factors. The results of that analysis are summarized in Table 5. As the chi square analysis of the adopted model in the logistic regression was statistically significant (X^2^ (9, *N* = 344) = 105.31, *p* < 0.01), the probability of an ADHD diagnosis is calculated better when the predictors are included in the equation. Results of the logistic regression analysis showed that controlling for gender is a significant predictor of ADHD diagnosis. In this relation, in comparison to girls, odds of diagnosing ADHD in boys were more than twice (OR = 2.45, *p* < 0.05) as high. Furthermore, as Fig. 1 indicates, this only applied approximately to vignettes 3 and 4 in which the percentages of receiving an ADHD diagnosis were 30.8% in boys and 17.5% in girls for vignette 3, and 15.9% in boys and 8.1% in girls for vignette 4. However, this discrimination was not obvious in vignette 2 (boys receiving an ADHD diagnosis: 77.5% and girls: 73.9%) and vignette 4 (boys receiving an ADHD diagnosis: 40% and girls: 33%).

**Table 5:** Results of the logistic regression predicting ADHD vs other diagnoses

Variables	B	SE	Odds Ratio	P
Gender of psychiatrist: female vs male	.263	.451	1.301	.560
Gender of vignette: boy vs girl	.897	.438	2.453	.041
Version of vignette				.000
1 vs. 2, 3, 4	4.417	.609	82.813	.000
2 vs. 1, 3, 4	1.632	.424	5.115	.000
3 vs. 1, 2, 4	1.122	.462	3.072	.015
Age of psychiatrist	.073	.053	1.076	.167
Years of job experience	−.117	.063	.890	.063
Helpfulness of DSM-IV	.016	.012	1.016	.191
Familiarity with DSM-IV	.011	.013	1.011	.368
Gender of child in case vignette **×** gender of psychiatrist	−.647	.622	.524	.298
Constant	−5.908	2.043	.003	.004

Note. DSM–IV.Diagnostic and Statistical Manual of Mental Disorders, fourth edition.

Moreover, we examined the overdiagnosis of ADHD separately for boy and girl vignettes. In this relation, the rate of false-positive diagnosis was 20.8% for girl vignettes, while the rate of false-negative ones was 6.5%, resulting in an odds ratio of 3.76 and a significant chi square (X^2^ (1, *N* = 171) = 4.86, *p* < 0.01). The same analysis for boys was also significant (X^2^ (1, *N* = 173) = 10.1, *p* < 0.01) with an odds ratio of 7.9, based on 29.3 and 5% rates of false-positive and false-negative diagnoses, respectively. These results imply that ADHD is being overdiagnosed in both boy and girl vignettes. However, by comparing the rates of false-positive diagnosis between boy and girl cases, our results demonstrate that boys are given significantly more false-positive diagnoses of ADHD (X^2^ (1, *N* = 258) = 5.87, *p* < 0.01).

### Influence of therapists’ characteristics on diagnostic decision

With respect to our third study question, namely to identify any gender bias by Iranian psychiatrists in diagnosing boys and girls with ADHD, we separately examined the occurrence of ADHD overdiagnosis by female and male therapists. In addition to the predictor variables, we included two interactions offered by Bruchmüller, Margraf and Schneider [21], namely the child’s gender in the case vignette^1–4^ X psychiatrist’s gender and child’s gender X type of case vignette (ADHD vs non-ADHD). The results of the logistic regression analysis indicated that the therapist’s gender and the interaction variables are not predictive of ADHD diagnosis (*p* = 0.23 and, *p* = 0.25, respectively).

Next, we examined the occurrence of psychiatrists’ overdiagnoses based on their gender on three levels. On level one, calculations were based on the overall case vignettes, i.e., irrelevant of child gender. The rates of false-positive and false-negative diagnoses were 25.8 and 4.65% for male therapists (OR = 7.12) and 24.6 and 6.9% for female therapists (OR = 4.3), respectively. Chi square was significant for both groups, implying that overdiagnosing ADHD is independent of the psychiatrist’s gender (female psychiatrist: X^2^ (1,*N* = 173) = 6.22,*p* < 0.01; male psychiatrist: X^2^ (1,*N* = 171) = 7.12, *p* < 0.01). On level two, we repeated the analysis once again by separately examining the occurrence of psychiatrists’ overdiagnosing ADHD in the boy and girl vignettes. Our results indicate that the female psychiatrists’ group rates of false-positive and false-negative diagnosis were 23.8 and 9.1% for girl and 25.4 and 4.8% for boy vignettes, respectively. Our analysis of the chi squares of girl vignettes was not significant (X^2^ (1,*N* = 85) = 2.2,ns, OR = 3.16), while the same statistic was significant for boy vignettes (X^2^ (1,*N* = 88) = 4.17,*p* < 0.01,OR = 6.8), showing that ADHD is overdiagnosed by female psychiatrists only in boy vignettes. In the male psychiatrists’ group, the rates of false-positive and false-negative diagnosis were 17.7 and 4.2% for girl vignettes, and 33.3 and 5.2% for boy vignettes, respectively. The analysis of chi squares of girl vignettes was not significant (X^2^ (1,*N* = 86) = 2.66,ns, OR = 4.9), while the same statistic was significant for boy vignettes (X^2^ (1,*N* = 85) = 5.89,*p* < 0.01,OR = 9.0). These results indicate that similar to female psychiatrists, ADHD is overdiagnosed by male psychiatrists only in boy vignettes. On level three, all calculations of previous levels were repeated while we excluded the answers from participants who had stated that either the information was insufficient to arrive at a diagnosis or that they only suspected ADHD. On this level, our results resemble those at previous levels and imply that both male and female psychiatrists are overdiagnosing ADHD, although only in boys.

### Influence of diagnosis on treatment recommendation

Addressing our fourth study question, we wanted to examine whether ADHD overdiagnosis reveals an impact on treatment recommendations. For each vignette psychiatrists were asked whether they would recommend any treatment at all and if so, whether they would choose psychotherapy, medication, or both. For vignettes 2–4, chi square tests were calculated to examine differences between treatment recommendations by the psychiatrists who made an ADHD diagnosis and those who did not. Only 172 therapists answered this section in the questionnaire. Our findings show that making an ADHD diagnosis significantly increases treatment recommendations entailing medication (X^2^ (1,*N* = 84) = 4.39,*p* < 0.01), whereas recommendations for psychotherapy were not significant (X^2^ (1,*N* = 79) = 4.27,*p* = 0.052). These findings indicate that ADHD overdiagnosis has a direct influence on medication prescriptions. In addition, comparing the treatment recommendations for ADHD cases (vignette 1), our findings show that 43 out of 65 psychiatrists answered the treatment-recommendation question in ADHD cases, and among them, 39 therapists prescribed methylphenidate – making it the most significant prescription for ADHD (v (1,*N* = 48) = 16.11,*p* = 0.052). In addition, 15 therapists recommended an adjunctive psychotherapeutic treatment (including cognitive behavioral therapy, psychoeducation and family counseling) and 5 therapists prescribed atomoxetine. None of the psychiatrists recommended mere psychotherapy for ADHD children.

## Discussion/conclusion

The Regarding our first study question, whether ADHD is overdiagnosed in Iran, we found that 25.2% of the psychiatrists diagnosed ADHD even though ADHD criteria had not been met, and 5.8% of therapists arrived at diagnoses other than ADHD even though the ADHD criteria had been fulfilled. As the false-positive rate of ADHD diagnosis was significantly higher than the false-negative one, our results imply the occurrence of ADHD overdiagnoses among Iranian psychiatrists, thus confirming our first hypothesis. Furthermore, 11.2% of psychiatrists (*n* = 29) made no clear diagnosis due to a lack of information, and another 5.4% of psychiatrists (*n* = 14) asserted a suspected ADHD diagnosis. When including those psychiatrists in our analyses, the calculated rate of false-positive diagnosis and thus rates of overdiagnosis rose further. These results are in line with those of Bruchmüller, Margraf & Schneider [21], as they also reported the occurrence of ADHD overdiagnoses among German therapists. They conclude that therapists rely on general heuristics and often fail to take diagnostic criteria into account. Supporting this is the review by Mertens, Cwik, Margraf and Schneider [11] on the overdiagnosis of disorders of childhood and adolescence that also demonstrates misdiagnoses of disorders for heuristic reasons rather than data-based decisions by diagnosticians.

Regarding our second study question as to whether the positive gender bias to boys’ disadvantage is also present in Iran, our results show that boys are also more likely to be falsely diagnosed with ADHD than girls. The girls’ false-positive rate was 20.8% and false-negative rate 6.5%, and the boys’ 29.3 and 6.5%, respectively. Those results indicate that ADHD is being overdiagnosed in both boys and girls, although the boys’ false-positive rate is significantly higher. This result contradicts the conclusion by Achenbach et al. [27] who established that although boys’ mean ASEBA and SDQ scores reported by parents and teachers in nearly every society are significantly higher than are those of girls, Iranian parents and teachers revealed no such gender differences. In this context, we suspect that Iranian parents and teachers tend to rate girls as high as boys on ADHD symptom measures. Accordingly, we had expected Iranian psychiatrists would rate girls and boys equally, and this would then have been reflected through similar ratings of the case vignettes. However, as we detected the same false-positive bias to the disadvantage of boys as Bruchmüller, Margraf and Schneider [21], it seems to be either that ADHD is perceived differently by health professionals in Iran, in this case by psychiatrists compared to teachers and parents (as in the Achenbach et al. [36] study) or that we are witnessing a change in ADHD symptom perceptions since 2008. As the meta-analysis by Yadegari et al. [37] revels a very high prevalence rate of ADHD of 12% (CI 95%: 9.0–15) in children aged 6 to 14 years in Iran, the latter could be the case. This 12% rate is also significantly higher than the worldwide rate of ~ 5% [1–3] and might further indicate that overall ADHD overdiagnosing in both genders is rampant, as our findings imply.

Regarding our third study question, namely to identify any gender bias among Iranian psychiatrists in diagnosing boys and girls with ADHD, we examined the occurrence of ADHD overdiagnosis by female and male psychiatrists separately and found that both male and female therapists overdiagnosed ADHD only in boys, a finding that does not concur with the results of Bruchmüller, Margraf & Schneider [21], as they reported ADHD overdiagnosis only by male therapists in boys and as an “unexpected finding with no obvious explanation.” Our study results are thus more in line with the assumption that both male and female psychiatrists do not always adhere to ADHD’s diagnostic criteria and instead most likely rely on heuristics as outlined above. In this context, the two main sources of the misdiagnosis are 1) similarities between the actual patient and an imaginary stereotypical ADHD patient; 2) prioritizing the diagnostic criteria differently. With respect to our results replicating a positive diagnostic bias to the disadvantage of boys, this might imply that a male patient exhibits significantly more similarities to a stereotypical ADHD patient than does a female patient. In this regard and as a suggestion for further studies, a deeper examination of the association between ADHD overdiagnosis and ADHD symptoms as well as ADHD subgroups would help us identify any specific criteria or subgroups associated with the occurrence of overdiagnosis. If we find evidence for such a hypothesis, we might be able to make diagnostic procedures more accurate by highlighting specific criteria or subgroups that make a misdiagnosis more probable. Furthermore, there are studies suggesting that there is a need for working through the ADHD criteria in a way that accounts for developmental, cultural, and gender differences [29, 38, 39].

Regarding our fourth study question, we wanted to investigate whether ADHD overdiagnosis has an influence on treatment recommendation. Confirming our expectation, our findings demonstrate that ADHD overdiagnosis had a direct impact on treatment recommendation, as psychiatrists who made an ADHD diagnosis in non-ADHD children prescribed medication more frequently than psychiatrists who did not. However, a similar psychotherapeutic-recommendation comparison was not significant. These findings are not fully in line with the study by Bruchmüller, Margraf & Schneider [21], as their results show that both psychostimulant and psychotherapeutic treatment recommendations were higher in the group of therapists that falsely declared an ADHD diagnosis. Explaining our findings, since all the participants in the present study were psychiatrists and psychologists were not included because they are not allowed to make a diagnosis due to the law of the Medical Council of the Islamic Republic of Iran, this factor might be a reason behind higher medication prescriptions and the lack of psychotherapy recommendations in this study. Moreover, there is solid evidence that not only has the overall prevalence rate of ADHD medication prescriptions increased during the last decade (e.g. [5, 19, 30, 40]), it is a rate also significantly higher for boys than girls [18, 20, 40]. In this regard and as our study confirmed, the occurrence of ADHD overdiagnoses is significantly linked to psychostimulant prescriptions, especially for boys. ADHD overdiagnosis has thus a strong influence on both patients and society as it is associated with the risk of unnecessary treatment and high health care costs.

There are currently no studies on the rate of methylphenidate prescription for ADHD in Iran and whether prescription rates have risen. Still, our results imply that patients with ADHD in Iran are receiving medical treatment (most frequently with methylphenidate) regardless of whether they are getting any psychotherapy.

With respect to potential bias in psychiatrists, post hoc analyses revealed that psychiatrists’ gender, age, years of job experience, as well as the interactions of the child’s gender in case vignette1–4 gender of psychiatrist do not play a significant role in predicting ADHD diagnoses (see Table 4). These results concur with those of Bruchmüller, Margraf & Schneider [21]. As both experienced and inexperienced therapists arrived at false-positive diagnoses, this is a matter of genuine concern. It is most likely that irrespective of psychiatrists’ job experience, their heuristic bias and subjective perception of the patient continue to influence their diagnoses.

Limitations of the study.

The first limitation of this study is the issue of case validity, as the vignettes in this study were adopted from the study by Bruchmüller, Margraf and Schneider [21] and are not fully compatible with Iranian culture. In this regard, the lack of the psychiatrist’s responsibility in decision-making based on the written vignettes is one source of such a limitation. In addition, compared to written descriptions of patients, psychiatrists in real situations can gather more information about a patient in real-life settings, which might facilitate the decision making process. However, as the vignettes are based strictly on the DSM-IV criteria and ICD-10 and as we would expect psychiatrists to strictly adhere to those criteria during the diagnostic procedure, decision-making should be even easier in such a situation than in real life settings [21].

Another issue that might be raised here is that arriving at a diagnosis in the field of mental disorders differs from diagnoses in other medical areas that have direct causal roots (like a bladder infection), as mental health disorders are much more prone to subjectivity (after all, a urine test will provide irrefutable evidence of a bladder infection [9]. However, to avoid the relativity accompanied by such subjectivity, we must rely on ADHD’s diagnostic guidelines and base diagnostic decisions on them if we want systematic measurements and assessments to prevail in our field and be able to validate interventions critically [36]. In addition, a reliable diagnosis serves as a valuable tool in improving interventions and in reducing probable overprescriptions of psychostimulants [8, 19].

The second limitation of the present study concerns the problem of generalizing results. As our study investigated the issue of overdiagnosis in a sample of Iranian psychiatrists from Tehran and Isfahan, our results might not transfer well to other cities in the country or to other countries in general. However, as our overall study, results, i.e., the occurrence of overdiagnosing ADHD and the significant role of patient gender in the psychiatrist’s diagnosis concur with the study of Bruchmüller, Margraf & Schneider [21] in their German sample and with regard to highlighting the presence of overdiagnosis in previous studies, we conclude that ADHD overdiagnosis is an issue in Iran and most other countries. Further investigation of this topic would reveal more details about the differences in overdiagnosis in other countries.

Finally, although at the time of conducting this study in 2019–2020 DSM-5 was already released, Iranian psychiatrists still base their diagnostic decision mostly on DSM-IV. A year after publication of DSM-5, the corresponding author of the present study, published a Persian review titled “Between DSM 5 & DSM -IV: a comparative glance on criteria” [41] as part of a project during master studies at Azad University of Science & Research Tehran. In this regard, this study was one of the first attempts to translate and summarize the differences between the diagnostic criteria of the manuals for the students and young clinicians. Furthermore, official Persian translations of the manual itself first appeared in 2015 and 2016 [42, 43]. After this, it was time for approving the reliability and validity of applying this manual for the Iranian population. However, results of a search analysis in a Persian academic data base, Noormag, and a global database, PubMed, showed that there are only 12 studies published in this regard, of which 10 belong to the years 2018–2021 (e.g. [44–46]). It seems due to the lack of such studies, Iranian clinicians still tend to rely on DSM-IV, for which there are still a bunch of qualified Persian diagnostic tools, though they seem to be using the DSM 5 as an adjunctive complementary tool. Furthermore, we believe that our adopted case vignettes and questionnaire from the seminal study of Bruchmüller, Margraf & Schneider [21] are still valid. There are two main subtle but important changes in criteria of ADHD in DSM-5 [47]: 1. To diagnose ADHD DSM-5 considers at least six symptoms of inattention or hyperactivity/impulsivity for children and adolescents and at least five symptoms for adults (more than 17 years of age), whereas DSM-IV considered for all the age ranges a minimum of 6 symptoms in each subtype. 2. Based on DSM-5, symptoms should have had an onset prior to 12 years of age, while the age of onset must be prior to 7 in DSM-IV. In addition, criterion C (pervasiveness) was changed from evidence of impairment to evidence of symptoms in two or more settings. Criterion D (impairment) now requires that functional impairments only need to “reduce the quality of social, academic or occupational functioning” instead of requiring that they be “clinically significant.” Criterion E (exclusionary conditions) no longer includes Autism Spectrum Disorder as an exclusionary diagnosis [47].

With regard to these changes, the case vignettes of the present study still result in the same diagnoses, i. e., in case vignette 1 the criteria of ADHD are fulfilled and in case vignettes 2–4 the criteria of ADHD are not fulfilled (failed to meet criteria b and c based on DSM-IV and criteria b based on DSM-5.). Therefore, revision of criteria in DSM-5 does not change our analysis and results.

## Conclusion

In conclusion, the present study systematically analyzed ADHD overdiagnoses among Iranian psychiatrists; our results reveal that ADHD is being overdiagnosed by both male and female psychiatrists, and that the child’s gender in the vignette also played a significant role, as boys were diagnosed with ADHD twice as often as girls in cases where ADHD should not have been diagnosed. In addition, our findings show that making a false-positive diagnosis of ADHD increases the frequency of psychostimulant treatment recommendations. Overall, our results imply that clinicians should strictly adhere to the criteria offered by ICD or DSM guidelines. In addition, to avoid the overdiagnosis of ADHD, we need to develop strategies to help overcome heuristic biases and reduce diagnostic error.

## Supplementary Information

**Additional file 1.**
