# Supplementary material for: ADHD overdiagnosis and the role of patient gender among Iranian psychiatrists
Source: BMC Psychiatry. 2021 Oct 19;21:514. doi: 10.1186/s12888-021-03525-3 (PMC8525031; doi:10.1186/s12888-021-03525-3)
Supplement: Supplementary file 1 — Additional file 1. [file 12888_2021_3525_MOESM1_ESM.docx]

***ADHD overdiagnosis and the role of patient gender among Iranian psychiatrists***

Ashkan Beheshti^a^, Mira-Lynn Chavanon^a^, Silvia Schneider^b^, Hanna Christiansen^a^

^a^ Department of Psychology, Child and Adolescent Clinical Psychology Group, Marburg University, Marburg, Germany

^b^ Department of Clinical Psychology, Child and Adolescent Psychology, Ruhr University Bochum, Germany

Short Title: ADHD overdiagnosis

Corresponding Author:

Ashkan Beheshti

Department of Psychology

Child and Adolescent Clinical Psychology Group

Gutenbergstr.18, 35037 Marburg

Marburg, 35037, Germany

Tel: 0049 (0) 6421 282 3668

E-mail: [beheshti@staff.uni-marburg.de](mailto:beheshti@staff.uni-marburg.de)

**Therapists’ Questionnaire**

The aim of this study is to get to know the approach of therapists in everyday practical life better in order to close the gap between theoretical research and practical approach. Therefore, the following questions raise which diagnosis you personally make and which therapy you would consider indicated in the described case.

As mentioned earlier, there is a large discrepancy between a case history and what you experience in personal contact with a patient. Therefore, in real contact you have the opportunity to inquire or possibly consult information from colleagues. Despite these differences, we would like to ask you to answer the following questions as if it were a real case.

**I What diagnosis would you give Sara?**

Please state the ICD-10 code number and the name of the disorder (e.g. F50.2 Bulimia nervosa).

**II How sure are you of your diagnosis?**

Please tick whether for example you are 25% or 80% sure.

|  | 0 | | 10 | | 20 | | 30 | | 40 | | 50 | | 60 | | 70 | | 80 | | 90 | | 100 | | % |
| --- | --- | --- | --- | --- | --- | --- | --- | --- | --- | --- | --- | --- | --- | --- | --- | --- | --- | --- | --- | --- | --- | --- | --- |
|  |  |  |  |  |  |  |  |  |  |  |  |  |  |  |  |  |  |  |  |  |  |  |  |
|  |  |  |  |  |  |  |  |  |  |  |  |  |  |  |  |  |  |  |  |  |  |  |  |
|  |  |  |  |  |  |  |  |  |  |  |  |  |  |  |  |  |  |  |  |  |  |  |  |
|  |  |  |  |  |  |  |  |  |  |  |  |  |  |  |  |  |  |  |  |  |  |  |  |

**III How sure are you that your colleagues would the exact same diagnosis?**

Please tick whether you are for example 25% or 80% sure.

|  | 0 | | 10 | | 20 | | 30 | | 40 | | 50 | | 60 | | 70 | | 80 | | 90 | | 100 | | % |
| --- | --- | --- | --- | --- | --- | --- | --- | --- | --- | --- | --- | --- | --- | --- | --- | --- | --- | --- | --- | --- | --- | --- | --- |
|  |  |  |  |  |  |  |  |  |  |  |  |  |  |  |  |  |  |  |  |  |  |  |  |
|  |  |  |  |  |  |  |  |  |  |  |  |  |  |  |  |  |  |  |  |  |  |  |  |
|  |  |  |  |  |  |  |  |  |  |  |  |  |  |  |  |  |  |  |  |  |  |  |  |
|  |  |  |  |  |  |  |  |  |  |  |  |  |  |  |  |  |  |  |  |  |  |  |  |

**IV Please indicate which treatment you would consider appropriate for Sara:**

For each alternative, please choose between "yes" and "no" and specify if necessary:

Psychotherapeutic treatment:  yes  no

If so, please specify:

Psychodynamic 

Behavioral therapy 

Other: ____________________

Drug treatment:  yes  no

If yes: with the following

Drug: _________________

Other support measures:  yes  no

If so, please specify:

Sports 

School support

(e.g. remedial classes etc.) 

Relaxation 

Other: ____________________

No intervention at all:  yes  no

**V Personal Data**

Please fill in and tick as appropriate.

1. Gender:  female  male

2. Age (in years): ____

3. I am:

 Physician

 Psychologist

 Pedagogue

 other: ___________________

4. I work:

 in a practice (____%)

 in a clinic (____%)

 in a counseling center (____%)

 other work area (____%)

5. My main therapeutic focus is:

(Please tick only one alternative)

 psychoanalytical  conversational psychotherapeutic

 cognitive-behavioral therapy  systemic

 depth psychology  gestalt therapy

 others: ___________________________

6. I have been practicing since (year, e.g. 1985): ______

7. How helpful do you find DSM-IV or ICD 10 diagnoses to be in your practical everyday life?

Not helpful at all very helpful

|  | 0 | | 10 | | 20 | | 30 | | 40 | | 50 | | 60 | | 70 | | 80 | | 90 | | 100 | | % |
| --- | --- | --- | --- | --- | --- | --- | --- | --- | --- | --- | --- | --- | --- | --- | --- | --- | --- | --- | --- | --- | --- | --- | --- |
|  |  |  |  |  |  |  |  |  |  |  |  |  |  |  |  |  |  |  |  |  |  |  |  |
|  |  |  |  |  |  |  |  |  |  |  |  |  |  |  |  |  |  |  |  |  |  |  |  |
|  |  |  |  |  |  |  |  |  |  |  |  |  |  |  |  |  |  |  |  |  |  |  |  |
|  |  |  |  |  |  |  |  |  |  |  |  |  |  |  |  |  |  |  |  |  |  |  |  |

8. How do you rate your ICD-10 or DSM IV knowledge?

Very limited comprehensive

|  | 0 | | 10 | | 20 | | 30 | | 40 | | 50 | | 60 | | 70 | | 80 | | 90 | | 100 | | % |
| --- | --- | --- | --- | --- | --- | --- | --- | --- | --- | --- | --- | --- | --- | --- | --- | --- | --- | --- | --- | --- | --- | --- | --- |
|  |  |  |  |  |  |  |  |  |  |  |  |  |  |  |  |  |  |  |  |  |  |  |  |
|  |  |  |  |  |  |  |  |  |  |  |  |  |  |  |  |  |  |  |  |  |  |  |  |
|  |  |  |  |  |  |  |  |  |  |  |  |  |  |  |  |  |  |  |  |  |  |  |  |
|  |  |  |  |  |  |  |  |  |  |  |  |  |  |  |  |  |  |  |  |  |  |  |  |

**Thank you for your cooperation!**

 yes, I agree that my data being used for goald of this study and would like to be informed about the results of the survey. My postal address / email address is:

Example of the Case Vignette (for a girl named Sara):

**1.1 ADHD fully fulfilled**

Sara, a ten year old girl, is very active and full of energy. She has troubles sustaining attention in one task for a longer time, and she is easily distracted by things around her. This leads to frequent difficulties at school. She often does not seem to listen when the teacher is saying something. She always loses her pens and leaves her books and school assignments anywhere. Furthermore, Sara is always on the move. She is constantly fidgeting with her feet or squirms in her seat. In school Sara often leaves her seat and walks around in the classroom or climbs on her seat even though she is expected to work quietly at her desk as all other children. She disturbs the other children not only with walking around. In class she also frequently interrupts other children when they are telling. She is constantly talking and has difficulty in awaiting turn. Therefore, her classmates do not like to be with her and exclude her when playing games. Sara becomes very angry in these situations and is offended. She blames her classmates for not letting her participate. Therefore, she is often alone at school. Also, at home, Sara’s agitated behavior often causes conflicts with her parents and her elder sister. She often avoids making schoolwork, and in daily activities like teeth brushing she often forgets what she just wanted to do. A pediatrician, to whom Sara’s parents went with her, observed no somatic diseases. Already in kindergarten Sara has been very inattentive and always on the move and therefore was ostracized when other children were playing.

**1.2 ADHD nonfulfilled: Symptom criteria A fulfilled, Criteria B (before age of 7) and C (impairment in two or more settings) not fulfilled**

Sara, a ten year old girl, is very active and full of energy. She has troubles sustaining attention in one task for a longer time, and she is easily distracted by things around her. This leads to frequent difficulties at school. She often does not seem to listen when the teacher is saying something. She always loses her pens and leaves her books and school assignments anywhere. Furthermore, Sara is always on the move. She is constantly fidgeting with her feet or squirms in her seat. In school Sara often leaves her seat and walks around in the classroom or climbs on her seat even though she is expected to work quietly at her desk as all other children. She disturbs the other children not only with walking around. In class she also frequently interrupts other children when they are telling. She is constantly talking and has difficulty in awaiting turn. Therefore, her classmates do not like to be with her and exclude her when playing games. Sara becomes very angry in these situations and is offended. She blames her classmates for not letting her participate. Therefore, she is often alone at school.

She often avoids making schoolwork, and in daily activities like getting her school assignments together she often forgets what she just wanted to do.

Her problems began about one year ago at the beginning of her fourth grade when she had to change her class and did not feel comfortable in her new class.

A pediatrician, to whom Sara’s parents went with her, observed no somatic diseases.

Outside of the school Sara does not have these problems. All in all, she obeys her parents well. She loves playing with her sister and also with her friends she gets along well.

**1.3 ADHD nonfulfilled: Symptom criteria A only partly fulfilled, Criteria B (before age of 7) and C (impairment in two or more settings) not fulfilled**

Sara, a ten year old girl, is very active and full of energy. She likes moving and talks and laughs a lot. She is popular in her class and is one of the children who are the “top dogs”. However, particularly with the math and biology teacher, her temperament causes problems. The teacher reports that Sara is very easily distracted by things around her. She often does not seem to listen when the teacher is saying something. She has troubles sustaining attention in one task for a longer time. Furthermore, Sara is always on the move. She is constantly fidgeting with her feet or squirms in her seat. This leads to remarkable problems in these two subjects. Because of these problems the teacher already contacted Sara’s parents. The problems began about one year ago at the beginning of the fourth grade when Sara’s class got this teacher for the first time. In other subjects the teachers also notice Sara’s temperament and her energy. However, it does not result in such manifest problems there. A pediatrician, to whom Sara’s parents went with her, observed no somatic diseases.

Outside of the school Sara does not have these problems. All in all, she obeys her parents well. She loves playing with her sister and also with her friends she gets along well.

**1.4 Generalized Anxiety disorder Criteria fulfilled. In this context also symptoms of agitation and restlessness, but no ADHD fulfilled**

Sara, a ten year old girl, is very restless, nervous and has difficulties in concentration. At school she often has problems in sustaining attention and following in class. She worries daily a lot about everyday occurrences and problems. For example, she is afraid that something could happen to her family. Furthermore, she is afraid of not passing the exams at school and of getting a severe illness. Her teacher repeatedly said she would have the feeling that Sara is always tense and seems to be restless and agitated. Furthermore, she would be very irritable and thin-skinned. Also, Sara herself reports having difficulties to relax and being “somehow always hyper”. She wishes becoming calmer and “getting the bad thoughts under control”. Questioned about the beginning of the difficulties, Sara’s parents report that she always had been an agitated and anxious child. However, since one year the problems would have reached the above reported dimension. A pediatrician, to whom Sara’s parents went with her, observed no somatic diseases. Sara herself says that she would be seriously affected by the reported difficulties. She wishes very much not to be so stressed, anxious and nervous anymore.
